# Postmortem Stability of Ebola Virus

**DOI:** 10.3201/eid2105.150041

**Published:** 2015-05

**Authors:** Joseph Prescott, Trenton Bushmaker, Robert Fischer, Kerri Miazgowicz, Seth Judson, Vincent J. Munster

**Affiliations:** National Institutes of Health, Hamilton, Montana, USA

**Keywords:** Ebola virus, viruses, outbreak, transmission, Ebola hemorrhagic fever, Ebola virus disease, postmortem stability, cynomolgus macaques, West Africa

## Abstract

The ongoing Ebola virus outbreak in West Africa has highlighted questions regarding stability of the virus and detection of RNA from corpses. We used Ebola virus–infected macaques to model humans who died of Ebola virus disease. Viable virus was isolated <7 days posteuthanasia; viral RNA was detectable for 10 weeks.

The ongoing outbreak of Ebola virus (EBOV) infection in West Africa highlights several questions, including fundamental questions surrounding human-to-human transmission and stability of the virus. More than 20,000 cases of EBOV disease (EVD) have been reported, and >8,000 deaths have been documented (*1*). Human-to-human transmission is the principal feature in EBOV outbreaks; virus is transmitted from symptomatic persons or contaminated corpses or by contact with objects acting as fomites (*2*). Contact with corpses during mourning and funeral practices, which can include bathing the body and rinsing family members with the water, or during the removal and transportation of bodies by burial teams has resulted in numerous infections (*3*).

Assessing the stability of corpse-associated virus and determining the most efficient sampling methods for diagnostics will clarify the safest practices for handling bodies and the best methods for determining whether a person has died of EVD and presents a risk for transmission. To facilitate diagnostic efforts, we studied nonhuman primates who died of EVD to examine stability of the virus within tissues and on body surfaces to determine the potential for transmission, and the presence of viral RNA associated with corpses.

## The Study

We studied 5 cynomolgus macaques previously included in EBOV pathogenesis studies and euthanized because of signs of EVD and viremia. Two animals were infected with EBOV-Mayinga and 3 with a current outbreak isolate (Makona-WPGC07) (*4*).

Immediately after euthanasia, multiple samples were collected: oral, nasal, ocular, urogenital, rectal, skin, and blood (pooled in the body cavity) swab samples and tissue biopsy specimens from the liver, spleen, lung, and muscle. Swabs were placed in 1 mL of culture medium and tissue samples were placed in 500 μL of RNAlater (QIAGEN, Valencia, CA, USA), or an empty vial for titration, before freezing at −80°C. Carcasses were placed in vented plastic containers in an environmental chamber at 27°C and 80% relative humidity throughout the study to mimic conditions in West Africa (*5*). At the indicated time points (<9 days for 2 animals and 10 weeks for 3 animals), swab and tissue samples were obtained and used for EBOV titration on Vero E6 cells to quantify virus or for quantitative reverse transcription PCR (qRT-PCR) (40 cycles) to measure viral RNA, as reported (*6*,*7*).

**Figure 1:**
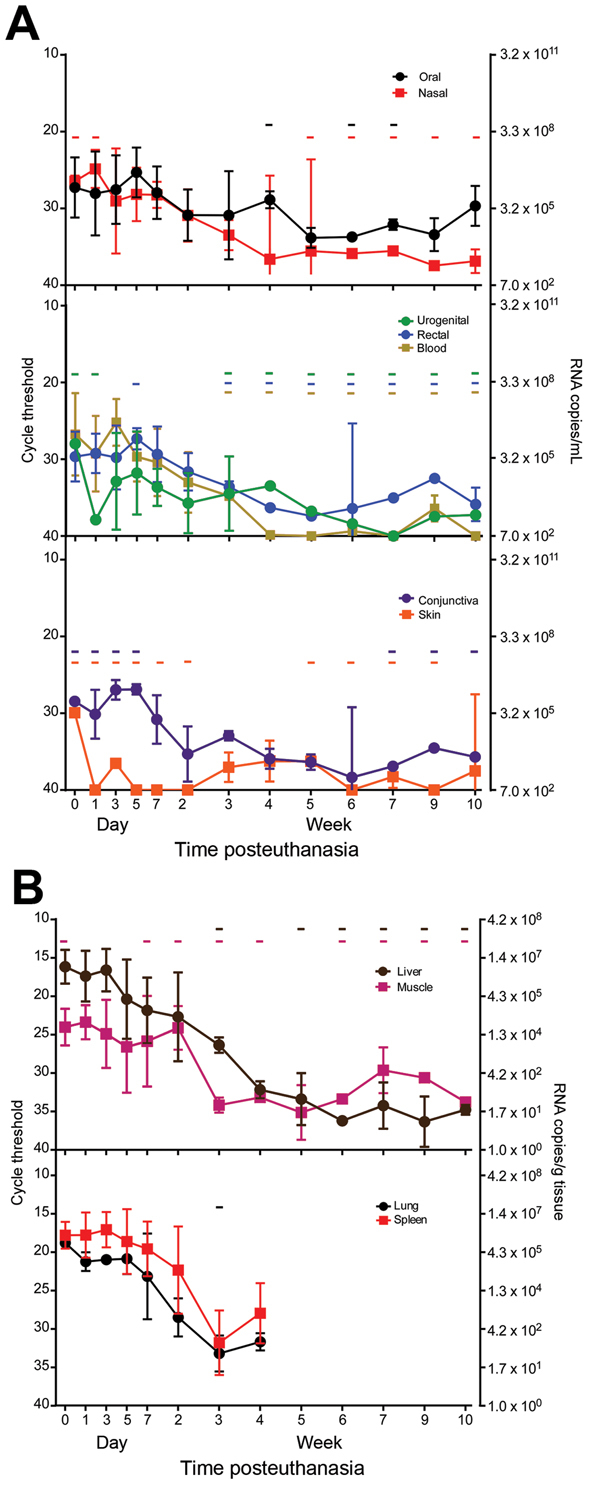
Presence and stability of Ebola virus RNA in deceased cynomolgus macaques. Swab (A) and tissue (B) specimen samples were obtained at the indicated time points, and viral RNA was isolated and used in a 1-step quantitative reverse transcription PCR with a primer/probe set specific for the nucleoprotein gene and standards consisting of known nucleoprotein gene copy numbers. Line plots show means of positive samples from 5 animals up to the 7 day time point and from 3 animals thereafter. Error bars indicate SD, and - indicates time points at which ≥1 animal had undetectable levels of viral RNA. Absence of a hyphen indicates that all animals had detectible levels of viral RNA.

Viral RNA was detectible in all swab samples and tissue biopsy specimens at multiple time points (Figure 1). For swab samples (Figure 1, panel A), the highest amount of viral RNA was in oral, nasal, and blood samples; oral and blood swab specimens consistently showed positive results for all animals until week 4 for oral specimens and week 3 for blood, when 1 animal was negative for each specimen type. Furthermore, oral swab specimens had the highest amount of viral RNA after the first 2 weeks of sampling, although after the 4-week sampling time point, some samples from individual animals were negative.

In all samples, RNA was detectable sporadically for the entire 10-week period, except for blood, which had positive results for <9 weeks. Tissue samples were more consistently positive within the first few weeks after euthanasia (Figure 1, panel B). All samples from the liver and lung were positive for the first 3 weeks, and spleen samples were positive for the first 4 weeks, at which time lung and spleen samples were no longer tested because of decay and scarcity of tissue. Muscle sample results were sporadic: a sample from 1 animal was negative at the 1-day time point and at several times throughout sampling.

**Figure 2:**
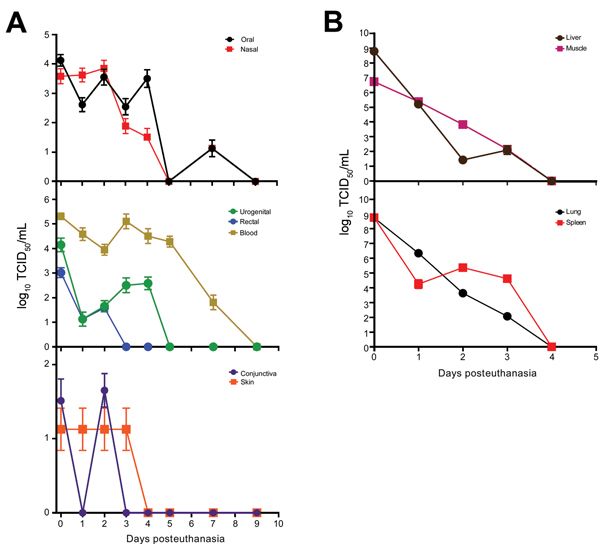
Efficiency of Ebola virus isolation from deceased cynomolgus macaques. Swab (A) and tissue (B) specimen samples were obtained at the indicated time points, and virus isolation was attempted on Vero E6 cells. Cells were inoculated in triplicate with serial dilutions of inoculum from swab specimens placed in 1 mL of medium or tissues homogenized in 1 mL of medium. The 50% tissue culture infectious dose (TCID_50_) was calculated by using the Spearman-Karber method (*8*). Line plots show means of positive samples from 5 animals to the day 9 time point. Error bars indicate SD.

Viable EBOV was variably isolated from swab from all sampling sites. Among blood samples, those from the body cavity had the highest virus titer (2 × 10^5^ 50% tissue culture infectious doses/mL) and longest-lasting isolatable virus (7 days posteuthanasia) (Figure 2, panel A). Consistent with the qRT-PCR results, for swab samples, oral and nasal sample titers were highest, followed by those for blood samples, and relatively high titers were observed <4 days posteuthanasia (Figure 2, panel B). Similar to the qRT-PCR experiments, virus titers were higher in tissue samples than in swab samples but were not as sustained; all tissue samples were positive at day 3 posteuthanasia but negative by day 4.

## Conclusions

The efficiency of detecting EBOV from corpse samples has not been systematically studied; this information is needed for interpreting results for diagnostic samples for epidemiologic efforts during outbreaks. We showed that viral RNA is readily detectable from oral and blood swab specimens for <3 weeks postmortem from a monkey carcass that was viremic at the time of death, in environmental conditions similar to those during current outbreak (*5*).

The stability of the target RNA used for RT-PCR is more robust than that of viable virus because degradation of any part of the genome (or proteins and lipids) would compromise the ability of the virus to replicate. Thus, the ability to isolate replicating virus in cell culture from postmortem materials was much less sensitive than detection of viral RNA by qRT-PCR. The sensitivity for quantitating infectious virus is probably lowered because of limitations in isolation efficiency on cell culture and necessary dilutions of tissues for homogenization for titration. Nonetheless, we detected viable virus <7 days posteuthanasia in swab specimens and 3 days in tissues, and showed that infectious virus is present at least until these times. Because virus titers decreased relatively sharply, despite sensitivity issues, it is unlikely that viable virus persists for times longer than we measured.

Humans who die of EVD typically have high levels of viremia, suggesting that most fresh corpses contain high levels of infectious virus, similar to the macaques in this study (*9*). Furthermore, family members exposed to EVD patients during late stages of disease or who had contact with deceased patients have a high risk for infection (*2*). The presence of viable EBOV and viral RNA in body fluids of EVD patients has been studied, and oral swabbing has been shown to be effective for diagnosis of EVD by RT-PCR compared with testing of serum samples from the same persons (*10**,**11*). However, detection limits for diagnostic swab samples are unknown for early phases of EVD, and blood sampling is probably more sensitive and reliable for antemortem diagnostics and should be used whenever possible, which has also been shown with closely related Marburg virus (*12*).

Although these studies included data from outbreak situations, they are limited in their sampling numbers, swabbing surfaces, and time course, and it is unknown how predictive they are for samples collected postmortem. It is essential to stress that swab samples should be obtained by vigorous sampling to acquire sufficient biologic material for testing, and development of a quality-control PCR target (housekeeping gene target) would be beneficial for sample integrity assessment, which is a limitation of this study.

In summary, we present postmortem serial sampling data for EBOV-infected animals in a controlled environment. Our results show that the EBOV RT-PCR RNA target is highly stable, swabbing upper respiratory mucosa is efficient for obtaining samples for diagnostics, and tissue biopsies are no more effective than simple swabbing for virus detection. These results will directly aid interpretation of epidemiologic data collected for human corpses by determining whether a person had EVD at the time of death and whether contact tracing should be initiated. Furthermore, viable virus can persist for >7 days on surfaces of bodies, confirming that transmission from deceased persons is possible for an extended period after death. These data are also applicable for interpreting samples collected from remains of wildlife infected with EBOV, especially nonhuman primates, and to assess risks for handling these carcasses.
